# The calpain system is associated with survival of breast cancer patients with large but operable inflammatory and non-inflammatory tumours treated with neoadjuvant chemotherapy

**DOI:** 10.18632/oncotarget.10066

**Published:** 2016-06-15

**Authors:** Sarah J. Storr, Siwei Zhang, Tim Perren, Mark Lansdown, Hiba Fatayer, Nisha Sharma, Renu Gahlaut, Abeer Shaaban, Stewart G. Martin

**Affiliations:** ^1^ Department of Clinical Oncology, Division of Cancer and Stem Cells, University of Nottingham, Nottingham University Hospitals NHS Trust, City Hospital Campus, Nottingham, NG5 1PB, UK; ^2^ Leeds Institute of Cancer Medicine and Pathology, St James's Institute of Oncology, St James's University Hospital, Leeds, LS9 7TF, UK; ^3^ Department of Breast Surgery, St James's University Hospital, Leeds, LS9 7TF, UK; ^4^ Breast Screening Unit Leeds/Wakefield, Seacroft Hospital, Leeds, LS14 6UH, UK; ^5^ Department of Histopathology, University Hospitals Birmingham NHS Foundation Trust, Queen Elizabeth Hospital, Queen Elizabeth Medical Centre and the University of Birmingham, Birmingham, B15 2TH, UK

**Keywords:** calpain, calpastatin, breast cancer, neoadjuvant chemotherapy, survival

## Abstract

The calpains are a family of intracellular cysteine proteases that function in a variety of important cellular functions, including cell signalling, motility, apoptosis and survival. In early invasive breast cancer expression of calpain-1, calpain-2 and their inhibitor, calpastatin, have been associated with clinical outcome and clinicopathological factors.

The expression of calpain-1, calpain-2 and calpastatin was determined using immunohistochemistry on core biopsy samples, in a cohort of large but operable inflammatory and non-inflammatory primary breast cancer patients treated with neoadjuvant chemotherapy. Information on treatment and prognostic variables together with long-term clinical follow-up was available for these patients. Diagnostic pre-chemotherapy core biopsy samples and surgically excised specimens were available for analysis.

Expression of calpastatin, calpain-1 or calpain-2 in the core biopsies was not associated with breast cancer specific survival in the total patient cohort; however, in patients with non-inflammatory breast cancer, high calpastatin expression was significantly associated with adverse breast cancer-specific survival (*P*=0.035), as was low calpain-2 expression (*P*=0.031). Low calpastatin expression was significantly associated with adverse breast cancer-specific survival of the inflammatory breast cancer patients (*P*=0.020), as was low calpain-1 expression (*P*=0.003).

In conclusion, high calpain-2 and low calpastatin expression is associated with improved breast cancer-specific survival in non-inflammatory large but operable primary breast cancer treated with neoadjuvant chemotherapy. In inflammatory cases, high calpain-1 and high calpastatin expression is associated with improved breast cancer-specific survival. Determining the expression of these proteins may be of clinical relevance. Further validation, in multi-centre cohorts of breast cancer patients treated with neoadjuvant chemotherapy, is warranted.

## INTRODUCTION

The calpain system is a family of calcium-activated cysteine proteases including the archetypal members’ calpain-1 and calpain-2, and their endogenous inhibitor calpastatin. Aberrant expression and activity has been described in numerous disease states, including Alzheimer's disease and cancer (reviewed in [1, 2]). These proteases function in numerous cellular activities, such as cytoskeletal remodelling, cellular signalling, survival and apoptosis (reviewed in [1]). The expression levels of calpain and calpastatin have been described in a number of tumour types, including pancreatic, ovarian and gastro-oesophageal [3–5]. In breast cancer, high calpain-2 expression is associated with adverse breast cancer-specific survival of patients with basal-like phenotype or triple receptor negative tumours [6]; high calpain-1 expression is associated with relapse-free survival of HER2 positive patients treated with adjuvant chemotherapy followed by trastuzumab [7]; and low calpastatin expression is associated with the presence of lymphovascular invasion [8]. In breast cancer, high calpain expression has been linked with poor prognosis which is thought to be due to elevated calpain activity promoting cellular survival and cytoskeletal remodelling, which in turn, can promote a more invasive phenotype. However, the roles for calpain are paradoxical, with studies showing its importance in regulation of both apoptosis and survival.

There are varying levels of evidence to suggest a role for altered calpain activity in regulating apoptosis in response to a variety of chemotherapy agents. Calpain can cleave autophagy related gene (Atg) 5, which is involved in the formation of autophagosomes. Evidence suggests that Atg5 cleaved by calpain is an important pro-apoptotic event in drug-induced apoptosis [9]. Calpain-1 and calpain-2 are both activated *in vitro* after 24 hours of paclitaxel treatment of non-small cell lung carcinoma cells and are responsible for some of the protease-mediated processing that occurs in paclitaxel-mediated cell death [10]. Furthermore, calpain can cleave Bax, a protein that translocates to the mitochondria upon induction of apoptosis, in promyelocytic leukaemia cells during drug induced apoptosis [11, 12]. In addition to altering apoptotic pathways in cancer cells, calpain has also been implicated in some of the toxicities associated with chemotherapy. Calpain activity has been implicated in cardiac injuries following doxorubicin treatment, both *in-vitro* and *in-vivo* [13, 14], however over-expression of calpastatin has also been shown to enhance doxorubicin-induced cardiac injuries through calpain inhibition [15]. In addition, calpain has been shown to degrade neuronal calcium sensor 1 (NCS-1) following paclitaxel treatment which is important in irreversible peripheral neuropathy [16]. Contradictory evidence suggests that calpain activity may play a role in survival following drug-induced stress. Calpain can proteolyse Myc to generate a transcriptionally inactive product that can delay colon cancer cell death following treatment with chemotherapy agents [17]. Much of the research describes the effects of calpain activation in different cancer cell types, and after various drug treatments. The perplexing nature of calpain proteolysis suggests important roles in numerous cellular activities that are dependent upon cellular context.

The aim of this study was to investigate the expression of calpain-1, calpain-2 and calpastatin in diagnostic, core biopsy specimens from women with large but operable primary breast cancer who were subsequently treated with neoadjuvant chemotherapy prior to surgical excision of residual tumour. Where matched surgically excised specimens were available, calpain-1, calpain-2 and calpastatin expression was also determined in these samples. Data was analysed against pathological response, patient survival and available clinicopathological data to determine the effect of this protease system on disease outcome.

## RESULTS

### Calpain system expression

**Figure 1:**
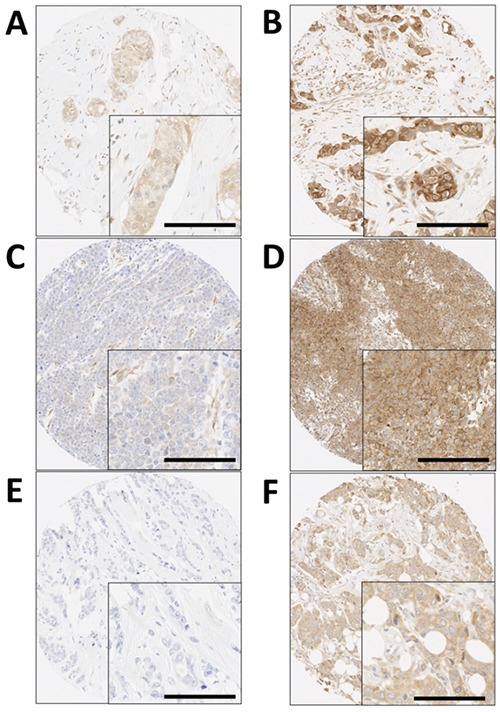
Representative photomicrographs following immunohistochemical staining of **A.** low calpastatin, **B.** high calpastatin, **C.** low calpain-1, **D.** high calpain-1, **E.** low calpain-2 and F. high calpain-2 staining. Photomicrographs are shown at 100× magnification with 200× magnification inset box where scale bar shows 100μm.

Calpain-1, calpain-2 and calpastatin expression displayed heterogenic cytoplasmic staining which varied from weak to strong with representative staining shown in Figure 1. Antibody specificity was demonstrated using Western blotting. Some cases were not assessed due to missing sample or insufficient representative tumour. Calpastatin had a median H-score of 140, ranging from 10-240 in the core biopsy samples, and 175, ranging from 103-250 in the surgically resected samples. Calpain-1 had a median H-score of 140, ranging from 0-240 in the core biopsy samples, and 150, ranging from 0-240 in the surgically resected samples. Calpain-2 had a median H-score of 160, ranging from 0-235 in the core biopsy samples, and 147.5, ranging from 0-183 in the surgically resected samples.

Spearman's rank order correlation test demonstrated no correlation for calpastatin (r^2^=0.155, *P*=0.253), calpain-1 (r^2^=0.178, *P*=0.193) or calpain-2 expression (r^2^=−0.075, *P*=0.561) in the core biopsy or the surgically excised specimen. Comparisons were made between core-biopsy and surgically excised specimens in non-inflammatory or inflammatory subgroups. In the non-inflammatory subgroup no correlation for calpastatin (r^2^=0.238, *P*=0.130), calpain-1 (r^2^=0.068, *P*=0.682) or calpain-2 expression (r^2^=−0.236, *P*=0.119) in the core biopsy or the surgically excised specimen was observed. In the inflammatory subgroup no correlation for calpastatin (r^2^=0.077, *P*=0.811), or calpain-2 expression (r^2^=−0.204, *P*=0.464) in the core biopsy or the surgically excised specimen was observed. Calpain-1 expression in the core biopsy or the surgically excised specimen was significantly correlated in the inflammatory subgroup (r^2^=0.680, *P*=0.011), although the correlation coefficient suggests this is of marginal biological relevance.

In the core biopsy samples, calpain-1 expression was correlated with calpastatin expression (r^2^=0.283, *P*=0.001); however, the correlation coefficient suggests this is of marginal biological relevance. Calpain-1 expression was not correlated with calpain-2 expression (r^2^=−0.004, *P*=0.965); calpain-2 expression was not correlated with calpastatin expression (r^2^=0.097, *P*=0.258). In the surgically excised specimens no correlations were observed in any of the pairwise comparisons of protein expression.

### Relationship between calpain system expression and clinicopathological variables

The expression of calpain-2 in the core biopsies was not associated with any clinicopathological variables that were determined at the time the core biopsy was taken (Table 1). High expression of calpastatin was associated with ER positive tumours (χ^2^=5.720, d.f.=1, *P*=0.017) and high calpain-1 expression was associated with PgR positive tumours (χ^2^=5.457, d.f.=1, *P*=0.019).

**Table 1:** Associations between the expression of calpastatin, calpain-1 and calpain-2 determined in core biopsy samples with clinicopathological variables also determined from the core biopsy samples

	Calpastatin			Calpain-1			Calpain-2		
	Low	High	*P* value	Low	High	*P* value	Low	High	*P* value
Lymph node status									
Negative	26 (28.6%)	18 (19.8%)	0.442	30 (35.3%)	12 (14.1%)	0.867	30 (33.0%)	15 (16.5%)	0.565
Positive	24 (26.4%)	23 (25.3%)		30 (35.3%)	13 (15.3%)		28 (30.8%)	18 (19.8%)	
Pathological response									
None/minimal	10 (6.6%)	2 (1.7%)	0.081	7 (6.4%)	2 (1.8%)	0.250	6 (5.1%)	6 (5.1%)	0.593
Partial	43 (37.1%)	43 (37.1%)		53 (48.6%)	29 (26.6%)		56 (47.9%)	31 (26.5%)	
Complete	11 (9.5%)	7 (6.0%)		15 (13.8%)	3 (2.8%)		12 (10.3%)	6 (5.1%)	
Inflammatory disease									
Non-inflammatory	49 (36.6%)	43 (32.1%)	0.350	58 (46.4%)	28 (22.4%)	0.932	64 (47.4%)	30 (22.2%)	0.109
Inflammatory	26 (19.4%)	16 (11.9%)		26 (20.8%)	13 (10.4%)		22 (16.3%)	19 (14.1%)	
Tumour grade									
1	3 (2.6%)	3 (2.6%)	0.825	5 (4.6%)	1 (0.9%)	0.596	3 (2.6%)	3 (2.6%)	0.365
2	29 (25.2%)	21 (18.3%)		29 (26.6%)	14 (12.8%)		27 (23.3%)	22 (19.0%)	
3	31 (27.0%)	28 (24.3%)		38 (34.9%)	37 (33.9%)		41 (35.3%)	20 (17.2%)	
ER status									
Negative	36 (26.9%)	17 (12.7%)	**0.017**	38 (30.4%)	13 (10.4%)	0.111	35 (25.9%)	19 (14.1%)	0.716
Positive	38 (28.4%)	43 (32.1%)		45 (36.0%)	29 (23.2%)		50 (37.0%)	31 (23.0%)	
PgR status									
Negative	40 (29.9%)	27 (20.1%)	0.297	48 (38.4%)	15 (12.0%)	**0.019**	46 (34.1%)	23 (17.0%)	0.362
Positive	34 (25.4%)	33 (24.6%)		35 (28.0%)	27 (21.6%)		39 (28.9%)	27 (20.0%)	
HER2 status									
Negative	39 (41.9%)	33 (35.5%)	0.885	50 (57.5%)	20 (23.0%)	0.587	45 (48.4%)	26 (28.0%)	0.717
Positive	11 (11.8%)	10 (10.8%)		11 (12.6%)	6 (6.9%)		13 (14.0%)	9 (9.7%)	

The *P* values are resultant from Pearson χ^2^ test of association or Fishers Exact when an asterisk is included. Significant *P* values are indicated in bold. ER is oestrogen receptor and PgR is progesterone receptor.

The expression of calpastatin and calpain-1 in the surgically excised samples was not associated with any clinicopathological variables determined on the surgically excised sample (Table 2). High calpain-2 expression was associated with inflammatory disease (χ^2^=6.792, d.f.=1, *P*=0.009) (Table 2). No association was observed between calpain or calpastatin expression and lymphovascular invasion when assessed in haematoxylin and eosin stained surgically resected specimens.

**Table 2:** Associations between the expression of calpastatin, calpain-1 and calpain-2 determined in surgically excised samples with clinicopathological variables also determined from the surgically excised samples

	Calpastatin			Calpain-1			Calpain-2		
	Low	High	*P* value	Low	High	*P* value	Low	High	*P* value
Lymph node status									
Negative	17 (37.0%)	2 (4.3%)	0.086*	11 (22.9%)	8 (16.7%)	0.675	12 (25.5%)	6 (12.8%)	0.869
Positive	17 (37.0%)	10 (21.7%)		15 (31.3%)	14 (29.2%)		20 (42.6%)	9 (19.1%)	
Pathological response									
None/minimal	8 (15.1%)	1 (1.9%)	0.418*	6 (10.2%)	4 (6.8%)	0.506*	8 (14.0%)	2 (3.5%)	0.295*
Partial	30 (56.6%)	14 (26.4%)		23 (39.0%0	26 (44.1%)		28 (49.1%)	19 (33.3%)	
Inflammatory disease									
Non-inflammatory	31 (56.4%)	12 (21.8%)	0.730*	25 (41.0%)	21 (34.4%)	0.334	32 (53.3%)	13 (21.7%)	**0.009**
Inflammatory	8 (14.5%)	4 (7.3%)		6 (9.8%)	9 (14.8%)		5 (8.3%)	10 (16.7%)	
Tumour grade									
1	3 (6.0%)	1 (2.0%)	0.774	1 (1.9%)	3 (5.8%)	0.227	2 (3.8%)	2 (3.8%)	0.841
2	14 (28.0%)	7 (14.0%)		8 (15.4%)	14 (26.9%)		15 (28.8%)	8 (15.4%)	
3	19 (38.0%)	6 (12.0%)		15 (28.8%)	11 (21.2%0		16 (30.8%0	9 (17.3%)	
ER status									
Negative	14 (26.4%)	5 (9.4%)	0.990	11 (19.3%)	10 (17.5%)	0.707	15 (27.3%)	6 (10.9%)	0.606
Positive	25 (47.2%)	9 (17.0%)		17 (29.8%)	19 (33.3%)		22 (40.0%)	12 (21.8%)	
PgR status									
Negative	19 (35.8%)	7 (13.2%)	0.934	16 (28.1%)	12 (21.1%)	0.234	20 (36.4%)	8 (14.5%)	0.504
Positive	20 (37.7%)	7 (13.2%)		12 (21.1%)	17 (29.8%)		17 (30.9%)	10 (18.2%)	
HER2 status									
Negative	37(71.2%)	14 (26.9%)	0.288*	29 (52.7%)	25 (45.5%)	0.473*	37 (68.5%)	16 (29.6%)	0.315*
Positive	0 (0.0%)	1 (1.9%)		0 (0.0%)	1 (1.8%)		0 (0.0%)	1 (1.9%)	
Size									
less than 2cm	14 (30.4%)	1 (2.2%)	0.070*	11 (22.9%)	6 (12.5%)	0.278	10 (21.3%)	6 (12.8%)	0.555
2cm or greater	20 (43.5%)	11 (23.9%)		15 (31.3%)	16 (33.3%)		22 (46.8%)	9 (19.1%)	

The *P* values are resultant from Pearson χ^2^ test of association or Fishers Exact when an asterisk is included. Significant *P* values are indicated in bold. ER is oestrogen receptor and PgR is progesterone receptor.

### Calpain system expression and patient survival

When plotted according to the Kaplan-Meier method with significance determined using the log-rank test, the expression of calpastatin, calpain-1 and calpain-2 in the core biopsies was not associated with breast cancer specific survival in the total patient cohort (*P*=0.470, *P*=0.790, *P*=0.174 respectively). Expression was separately analysed in both the non-inflammatory cases and inflammatory breast cancer, with the latter having a significantly worse breast cancer-specific survival (*P*=1×10^−5^) in this cohort.

**Figure 2:**
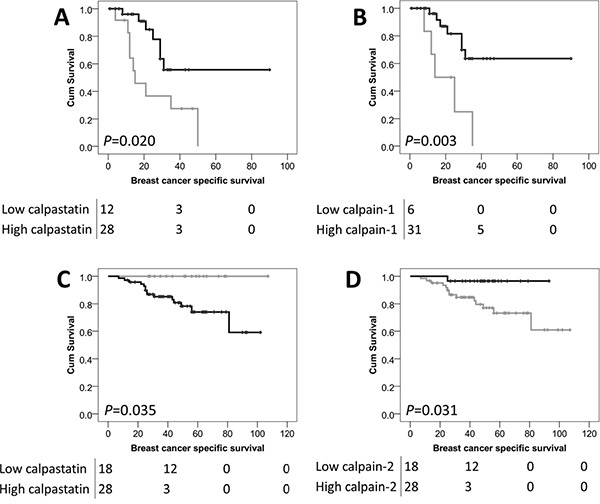
Kaplan-Meier analysis of breast cancer specific survival showing the impact of high (black line) and low (grey line) calpastatin **A.** and calpain-1 **B.** in patients with inflammatory breast cancer and calpastatin **C.** and calpain-2 **D.** expression in patients with non-inflammatory breast cancer in the core biopsy samples. Significance was determined using the log-rank test and the numbers shown below the Kaplan-Meier survival curves are the number of patients at risk at 0, 40, 80 and 120 months.

In the core biopsies, high calpastatin expression was significantly associated with adverse breast cancer-specific survival (*P*=0.035) in the non-inflammatory breast cancer cases (Figure 2C), whereas low calpastatin was significantly associated with adverse survival (*P*=0.020) in the inflammatory breast cancer cases (Figure 2A). Low calpain-1 expression was associated with adverse survival in inflammatory breast cancer cases (*P*=0.003) (Figure 2B) and low calpain-2 was associated with adverse survival in the non-inflammatory breast cancer cases (*P*=0.031) (Figure 2D).

In the surgically excised cases, expression of calpastatin, calpain-1 and calpain-2 was not associated with breast cancer specific survival in the total patient cohort (*P*=0.347, *P*=0.428, and *P*=0.220 respectively); no association with survival of the inflammatory or non-inflammatory subgroups was observed.

The association between protein expression and patient survival in the inflammatory and non-inflammatory subgroups was explored further. All traditional prognostic variables were individually tested for their association with survival in the inflammatory and non-inflammatory patient subgroups using the Kaplan-Meier method with significance determined using the log-rank test. In the inflammatory subgroup none of the traditional prognostic variables were associated with patient survival including patient age, histological grade, tumour size, lymph node status and ER, PgR or HER2 receptor status (with individual Kaplan-Meier statistics of *P*=0.450, *P*=0.188, *P*=0.709, *P*=0.239, *P*=0.814, *P*=0.194 and *P*=0.953 respectively). Multivariate Cox regression was performed using these potential confounding factors, despite them not individually being associated with survival; calpastatin expression remained significantly associated with the survival of patients with inflammatory disease (Hazard ratio (HR)= 0.36, 95% confidence interval (CI)= 0.001-0.896, *P*=0.043) (Table 3). Calpain-1 expression was not independently associated with survival when potential confounding factors were included in the analysis.

**Table 3:** Cox proportional hazards analysis for overall survival for calpastatin expression in breast cancer patients with large but operable inflammatory disease

	*P* value	Exp(B)	95.0% CI for Exp(B)	
			Lower	Upper
Calpastatin	0.043	0.036	0.001	0.896
Tumour grade	0.125	0.046	0.001	2.351
tumour size	0.549	3.142	0.074	132.603
Patient age	0.480	0.285	0.009	9.329
ER status	0.292	0.013	0.000	40.877
PgR status	0.267	20.462	0.099	4232.348
HER2 status	0.376	1.712	0.521	5.626

Exp (B) is used to denote hazard ratio and 95% CI is used to denote 95% confidence interval.

In the non-inflammatory patient subgroup none of the traditional prognostic variables were associated with patient survival including patient age, histological grade, tumour size, lymph node status and ER, PgR or HER2 receptor status (with individual Kaplan-Meier statistics of *P*=0.429, *P*=0.935, *P*=0.244, *P*=0.648, *P*=0.568, *P*=0.141 and *P*=0.892 respectively). Multivariate Cox regression was performed using these potential confounding factors, despite them not being individually associated with survival; neither calpastatin nor calpain-2 expression were independently associated with survival in this analysis.

## DISCUSSION

The expression of calpain-1, calpain-2 and calpastatin was determined in diagnostic core biopsy samples taken from patients with large but operable breast cancer or locally advanced breast cancer who subsequently received neoadjuvant chemotherapy prior to surgical resection. In addition, matched samples from surgically excised specimens were available and were also assessed for calpain-1, calpain-2 and calpastatin expression. This study demonstrates that high calpastatin expression in the diagnostic core biopsy was significantly associated with improved survival in patients with inflammatory breast cancer, while the reverse was observed in non-inflammatory cases. In addition, low calpain-2 expression was associated with adverse survival in non-inflammatory breast cancer patients and low calpain-1 expression was associated with adverse survival in inflammatory breast cancer patients.

The calpain system has previously been shown to be associated with the prognosis of breast cancer patients with early invasive disease, when determined on surgically excised specimens prior to adjuvant treatments. Studies have shown that high calpain-2 expression is associated with adverse breast cancer-specific survival in basal-like and triple-negative phenotype breast cancers [6], high calpain-1 expression and adverse relapse free survival in HER2 positive breast cancer patients treated with adjuvant chemotherapy and trastuzumab and high calpastatin expression with the absence of lymphovascular invasion determined by immunohistochemistry [7, 8]. However, in the current study we have shown in large but operable breast cancer, where expression of the calpain system was determined in core biopsies taken prior to neoadjuvant chemotherapy and in tissue from surgical resection, that high calpastatin expression and high calpain-1 is significantly associated with improved breast cancer-specific survival in patients with inflammatory cancer and that low calpastatin or high calpain-2 expression is significantly associated with improved breast cancer-specific survival in patients with non-inflammatory cancer. Such results suggest that the calpain system plays differing roles in different steps of tumour progression and in response to therapy pre- and post- tumour excision. Perhaps the most interesting observation is that of calpastatin expression, whereby high expression is associated with improved survival of inflammatory breast cancer and high expression is associated with adverse survival of non-inflammatory breast cancer. This observation suggests that the role that calpastatin plays in these two breast cancer types is distinct and important.

The calpain system has been shown to function in numerous cellular functions, including cytoskeletal remodelling, cell signalling, survival and apoptosis, and we have previously hypothesised that these paradoxical functions depend upon the context of calpain activation. In this study, we have measured calpain system expression, rather than resultant calpain activity in core biopsy samples from patients subsequently treated with neoadjuvant chemotherapy and surgical resection. As calpain activity is closely linked to apoptosis, it may be that calpain activity is important in tumours that will be treated with neoadjuvant chemotherapy. The reverse may be true when early invasive tumours are surgically resected and patients treated with various different therapies, including radiotherapy and adjuvant chemotherapy. We cannot predict the levels of calpain activity from the expression of the calpain system, as various factors are involved with the activation of the protease. It would be of interest to determine calpain activity, which may be possible using antibodies against the products of calpain proteolysis; however such reagents would require validation to determine their use in malignant tissue [18, 19].

Calpain and calpastatin have been shown to be linked with the process of lymphovascular invasion in previous tissue based and *in-vitro* studies [8, 20]. In this study, no association was observed between calpain or calpastatin expression and lymphovascular invasion determined in the surgically resected sample, which may be due to determination being made on haematoxylin and eosin stained specimens over those stained with antibodies against lymphatic or blood endothelial cell specific markers. Inflammatory breast cancer can be associated with exaggerated frequencies of lymphovascular invasion and lymph node involvement [21], however in the inflammatory subset, no association was observed between lymphovascular invasion and the expression of calpain or calpastatin. There was also no association between lymphovascular invasion and inflammatory disease (χ^2^=1.939, d.f.=1, *P*=0.164); the presence of inflammatory disease was significantly associated with survival of patients (*P*=1.0×10^−5^) as was lymphovascular invasion (*P*=0.004). These patients have all been treated with up to eight cycles of neoadjuvant chemotherapy which is likely to have affected these findings.

This study has demonstrated that low expression of calpain-2 and high expression of calpastatin determined in diagnostic core biopsy samples is significantly associated with adverse breast cancer-specific survival in non-inflammatory large but operable primary breast cancer. Furthermore, low expression of calpastatin and calpain-1 is significantly associated with adverse breast cancer-specific survival in inflammatory large but operable primary breast cancer. In addition to the association with survival of these patients, high calpastatin expression is associated with ER positive tumours and high calpain-1 expression is associated with PgR positive tumours.

These findings may have important implications when investigating calpain and calpastatin expression in breast cancer as this data suggests that the presence of calpain, presumably active calpain, is beneficial for tumours treated with chemotherapy prior to surgery and that the traditional view that calpain activity in breast cancer is clearly associated with adverse patient prognosis may be setting dependent. Importantly this may suggest that future calpain inhibition strategies as a method of therapeutic intervention in breast cancer may require important treatment caveats imposed dependent upon the patient group and planned treatment regimen.

In conclusion, low calpain-2 and high calpastatin expression are associated with adverse breast cancer-specific survival in non-inflammatory large but operable breast cancer treated with neoadjuvant chemotherapy, and determining the expression of these important proteins may be of clinical relevance. Furthermore, low calpain-1 and calpastatin expression are associated with adverse survival of patients with inflammatory large but operable breast cancer. As part of future research, these results require validation in multi-centre cohorts of locally advanced breast cancer patients treated with neoadjuvant chemotherapy.

## MATERIALS AND METHODS

### Clinical samples

This study is reported in accordance with REMARK criteria. Ethical approval was granted under the title ‘Studies of the biological significance of breast cancer subtype’ (Leeds (East) REC no: 06/Q1206/180). This study utilised 143 4μm thick whole mount sections of formalin fixed paraffin embedded (FFPE) core biopsy specimens and 4μm sections of a tissue microarray of surgically excised FFPE specimens from 68 matched patients. Comprehensive clinical data, including chemotherapy regimen, type of surgery and imaging characteristics (mammography and MRI) were collected.

Patients were treated at the Leeds Teaching Hospitals NHS Trust in the period between 2005 and 2009 and had long term follow-up information available. The median survival time was 100 months; the median follow-up of patients alive (n=76) was 49 months (range 1-107) as calculated by the reverse Kaplan-Meier method. Patients included in this study were those with inflammatory or non-inflammatory breast cancer where primary surgery was one of the options considered; but due to the anatomical, pathological or molecular characteristics of the tumour, the patient would also be an obligate candidate for cytotoxic chemotherapy. The decision was made with the patient to use chemotherapy as the primary treatment (neo-adjuvant chemotherapy) so that chemotherapy drugs could be tailored to response, and with the hope that following response to chemotherapy surgery may be less radical. For patients who failed to respond, or those patients with residual invasive disease (no response/partial pathological response), slides were reviewed and a representative block was selected and marked for tissue microarray construction as per the published guidance [22].

Initial treatment was anthracycline based chemotherapy and response was monitored by serial MRI scanning at baseline and repeated after every two cycles of treatment according to standard practice; chemotherapy was switched to docetaxel at the point when the response was considered inadequate. The modified response criteria was as follows; complete response was the complete radiological absence of previously documented lesions; almost complete response was the complete radiological absence of all previously documented lesions and a blush of residual enhancement; partial response was a greater than 50% reduction in diameter of the of the main tumour bulk or 25-50% reduction in the diameter of the main tumour bulk and improved enhancement curve; minimal response was 25-50% reduction in main tumour bulk and no improvement in enhancement curve; stable response was no change to appearance of tumour and progression was defined as progressive lesions or 25% increase in tumour bulk. Trastuzumab was introduced in patients who were HER2 positive at the time of switch to docetaxel chemotherapy. All patients received between 6 and 8 cycles of chemotherapy which was followed by surgical treatment of residual disease, the extent of surgery being determined by MRI scanning and multi-disciplinary team discussion. Following surgical excision patients received adjuvant endocrine therapy, trastuzumab, and radiotherapy according to standard practice.

The median age of the patients was 48 years (ranging from 23-83 years), 49.5% of cases were lymph node negative (46/93), 68.6% of patients had non-inflammatory disease (94/137), 30.4% of patients had inflammatory disease (43/137) and 52.5% of patients had grade III disease at the time of core biopsy (61/118); where patient number does not total 143 information was not available for the remaining patients. Detailed clinicopathological data was recorded at the time of core biopsy and is shown in Table 1. Patients were assessed and managed in a standardised manner according to their clinical history and tumour characteristics. Breast cancer-specific survival was defined as the time from diagnosis to death from breast cancer. Histopathological features were reported as complete pathological response, where no residual invasive tumour was observed; partial response, where evidence of response to therapy was observed; and no/minimal evidence of response to therapy [23].

### Immunohistochemistry

Tissue microarray construction has been described previously [24]. Slides were deparaffinised in xylene, followed by rehydration in ethanol and water. Antigen retrieval was performed in 0.01mol L^−1^ sodium citrate buffer (pH6.0) in a microwave for 20 minutes, with 10 minutes at 750W and 10 minutes at 450W. Immunohistochemistry was performed using a Novolink Polymer Detection kit (Leica) according to the manufacturers’ instructions. Briefly, Peroxidase Block solution was added to tissues following antigen retrieval, and tissue was then washed with Tris-buffered saline before incubation with Protein Block solution. Primary antibodies against calpain-1 (1:1000; Santa Cruz Biotechnology clone P-6), calpain-2 (1:2500; Chemicon rabbit polyclonal AB1625) and calpastatin (1:40000; Chemicon clone PI-11) were incubated on tissue for one hour at room temperature, with antibody specificity determined by Western blotting. Following antibody incubation, tissues were washed and subject to incubation with Post Primary solution, then washed and incubated with Novolink Polymer solution. Immunohistochemical reactions were developed using 3,3′ diaminobenzidine as the chromogenic substrate and sections were counterstained with haematoxylin. After staining, tissues were dehydrated and fixed in xylene prior to mounting with DPX (Sigma). Breast tumour composite sections comprised of grade 1 and 2 early stage invasive tumours were included as positive and negative controls, where in the negative control primary antibody was omitted during each staining run.

Assessment of staining was conducted at x200 magnification using a Nikon Eclipse 80i microscope for core biopsy specimens, or following scanning the slides using a Nanozoomer Digital Pathology Scanner (Hamamatsu Photonics) for the TMA. Staining was assessed semi-quantitatively using an immunohistochemical *H*-score, where staining intensity was assessed as none (0), weak (1), medium (2), and strong (3) over the percentage area of each staining intensity. *H*-scores were calculated by multiplying the staining intensity by the staining area and resulted in *H*-scores ranging from 0 to 300. Both core biopsy and the TMA of surgically excised specimens had greater than 30% of the cases scored by two independent assessors, blinded to each other's scores and the clinical outcome of the patients. The single measures intra-class correlation efficient was used to determine the level of concordance between scorers, which was above 0.7 for all markers assessed, indicating good concordance.

### Statistical analyses

Statistical analyses were performed using SPSS 22.0 software and protein expression was dichotomised into high and low expression based on breast cancer-specific survival using X-tile software which allows for non-biased cut point generation [25]. Relationships between categorised protein expression and clinicopathological variables were determined using Pearson Chi Square test of association (χ^2^) where there were more than two variables, or Fishers Exact test in a 2×2 table when a cell count less than 5 was observed. Overall disease-specific survival curves were plotted according to the Kaplan-Meier method with significance determined using the log-rank test. Spearman rank order correlations were used to assess the correlation between protein-protein expression. All differences were deemed significant at the level of *P*<0.05.
